# Supplementary material for: Physical Activity Behavior, Motivation and Active Commuting: Relationships with the Use of Green Spaces in Italy
Source: Int J Environ Res Public Health. 2022 Jul 28;19(15):9248. doi: 10.3390/ijerph19159248 (PMC9367901; doi:10.3390/ijerph19159248)
Supplement: Supplementary file 1 [file ijerph-19-09248-s001.zip › ijerph-1820356-supplementary.pdf]

Figure S1. Park and Recreation Users Survey Anonymous Questionnaire

Age______ **Sex**: ☐ Male ☐ Female **Weight**______ kg **Height**________ cm

Education level: ☐ Middle school ☐ High school ☐ Bachelor's degree ☐ Master's degree ☐ PhD

Occupation: ☐ Student ☐ Employed ☐ Unemployed ☐ Retired

Q1. How do you usually reach the park? ☐ Walking ☐ Running ☐ Bicycle ☐ Vehicle (car, moto, bus) ☐ Other______

Q2. If you have reached the park on foot and/or run and/or bike how many minutes you have employed to reach the park? _____________

Q3. From 1 (very easy) to 10 (very hard) how tired are you when you reach the park?_____________

Q4. How far (in meters, more or less) is your home from the park? _____________________

Q5. Do you go to the park to practice PA? ☐ yes ☐ no

Q6. If you do not practice PA at the park, why do you go to the park? ____________

Q7. If you go to the park to practice PA, what kind of exercise/activity are you usually into?

☐ Light running ☐ Walking ☐ Outdoor fitness equipment ☐ Stretching ☐ Yoga ☐ Bicycle ☐ Basketball ☐ Football ☐ Volleyball ☐ Skating ☐ Nordic Walking ☐ Other______

Q8. How many times per week do you go the park ?_________________

Q9. How many hours of PA do you practice in the park at a time? ___________

Q10. If there wasn't this park, would you have practiced PA in an indoor environment?
☐ Yes ☐ No ☐ I do not know

If yes, by how much?

☐ Once a week ☐ Two times a week ☐ Three times a week ☐ Four times a week

☐ Five times a week ☐ Everyday

Q11.Where do you like to exercise the most? ☐ Indoor (from 1 to 5) ……… ☐ Outdoor (from 1 to 5)……….

☐ 1. Very little ☐ 2. Little ☐ 3. Enough ☐ 4. Satisfied ☐ 5. Very satisfied

Q12. If you practice indoor exercise, in which type of indoor environment?

☐ gym ☐ home ☐swimming pool

Q13. Are you satisfied with this park? (a score from 1 to 5)

☐ 1. Very little ☐ 2. Little ☐ 3. Enough ☐ 4. Satisfied ☐ 5. Very satisfied

Q14. Evaluate the follow sentence: After doing PA in the park, I feel more energetic (1 means totally disagree, 5 totally agree) ☐ 1 ☐ 2 ☐ 3 ☐ 4 ☐5

Q15. Evaluate the follow sentence: After visiting the park, I feel more peaceful (1 means totally disagree and 5 totally agree) ☐ 1 ☐ 2 ☐ 3 ☐ 4 ☐5
